# Single-Cell Atlas of Adult Testis in Protogynous Hermaphroditic Orange-Spotted Grouper, *Epinephelus coioides*

**DOI:** 10.3390/ijms222212607

**Published:** 2021-11-22

**Authors:** Xi Wu, Yang Yang, Chaoyue Zhong, Tong Wang, Yanhong Deng, Hengjin Huang, Haoran Lin, Zining Meng, Xiaochun Liu

**Affiliations:** 1 State Key Laboratory of Biocontrol, Guangdong Province Key Laboratory for Improved Variety Reproduction of Aquatic Economic Animals, Institute of Aquatic Economic Animals, School of Life Sciences, Sun Yat-Sen University, Guangzhou 510275, China; wuxi577@126.com (X.W.); yangy595@mail2.sysu.edu.cn (Y.Y.); zhongchy9@mail2.sysu.edu.cn (C.Z.); sysuwangtong@163.com (T.W.); dengyh56@mail2.sysu.edu.cn (Y.D.); huanghj75@mail2.sysu.edu.cn (H.H.); lsslhr@mail.sysu.edu.cn (H.L.); mengzn@mail.sysu.edu.cn (Z.M.); 2 Southern Marine Science and Engineering Guangdong Laboratory (Zhuhai), Zhuhai 519000, China

**Keywords:** single-cell transcriptome, testis, spermatogenesis, protogynous, orange-spotted grouper

## Abstract

Spermatogenesis is a process of self-renewal and differentiation in spermatogonial stem cells. During this process, germ cells and somatic cells interact intricately to ensure long-term fertility and accurate genome propagation. Spermatogenesis has been intensely investigated in mammals but remains poorly understood with regard to teleosts. Here, we performed single-cell RNA sequencing of ~9500 testicular cells from the male, orange-spotted grouper. In the adult testis, we divided the cells into nine clusters and defined ten cell types, as compared with human testis data, including cell populations with characteristics of male germ cells and somatic cells, each of which expressed specific marker genes. We also identified and profiled the expression patterns of four marker genes (*calr*, *eef1a*, *s100a1*, *vasa*) in both the ovary and adult testis. Our data provide a blueprint of male germ cells and supporting somatic cells. Moreover, the cell markers are candidates that could be used for further cell identification.

## 1. Introduction

In vertebrates, with high transcriptional activity, the core function of the testis in sperm production is conserved across kingdoms. The testis primarily comprises male germ cells, including spermatogonial stem cells (SSCs), spermatogonia (SG), spermatocytes (SCs), spermatids (STs), and spermatozoa (SZ). During spermatogenesis, SSCs begin to proliferate, differentiate, and undergo meiosis, finally forming functional SZ (motile and fertile), followed by a series of post-testicular maturation processes [1,2]. SSCs differentiate into SG, which then proliferate and differentiate into SCs. The SCs subsequently enter meiosis to form haploid STs. Eventually, the transformation of STs to mature SZ requires significant physical and structural restructuring of the cells, leading to the production of haploid gametes. Moreover, there are many somatic cells in the testis, such as Leydig cells, Sertoli cells, endothelial cells, and macrophages. Male germ cells cannot differentiate successfully without the support of specialized somatic cells. These non-spermatogenic cells create a closed microenvironment that provides conditions for the self-renewal, development, and differentiation of spermatogenic cells, which are essential for the persistent production of SZ. In the testicular interstitial tissue, the main function of Leydig cells is to produce testosterone for the maintenance of spermatogenesis [3,4]. Sertoli cells maintain the function of male germ cells, provide paracrine support and a blood testosterone barrier for germ cells, and synthesize growth factors and hormone receptors [5]. Macrophages, which are frequently associated with the vasculature, have an important role in spermatogonial development [6,7]. However, the communication between somatic cells and germ cells still needs further research. Therefore, detailed information of every cell type needs to be revealed to analyze the reproductive physiology of testis and the corresponding molecular mechanism.

Recently, a new technology, single-cell transcriptome-sequencing, has overcome the homogenization of various cell types in the same tissue and yields the molecular characteristics of every cell type. Depending on its high throughput and accuracy, it can provide comprehensive profiles and unexpected insights when applied in many fields [8]. In the human testis, complete spermatogenesis and corresponding transcriptional signatures were revealed by single-cell RNA sequencing (scRNA-seq) [9]. By comparing gene expression profiles of cells from neonatal and adult testes, the developmental trajectories of germ and somatic cell types were also defined [10]. When comparing the spermatogenesis of humans, macaques, and mice, the similarities and differences of the differential markers, and potential regulators in meiosis were revealed [11]. In sheep testis, all cells and numerous stage-specific marker genes were identified through scRNA-seq [12]. However, in vertebrates, there have been few studies that have applied scRNA-seq to spermatogenesis in non-mammals.

In contrast to that in mammals, there are various patterns of sexual determination in teleosts, including gonochorism and hermaphroditism (protogynous, protandrous, and synchronous), which lead to a more complicated mechanism of gonadogenesis [13]. The orange-spotted grouper, a protogynous hermaphroditic fish, undergoes sex reversal from female to male during its life history, and it has been regarded a good fish model to study sex differentiation and sex reversal [14]. Therefore, it is important to acquire transcriptome information from every cell type in the testis to explore the mechanism of gonadogenesis, gonad differentiation, and sex reversal. In our previous study, laser-captured-microdissection (LCM) sequencing was used to obtain the specific transcriptome information of male germ cells in orange-spotted grouper. The differences among various male germ cells were compared, and many male-specific marker genes were identified, which provided initial insights into the spermatogenesis of orange-spotted grouper [15]. With the development of sequencing technology, it is now possible to use more convenient and accurate approaches to assess germline and somatic cell transcriptional profiles. Therefore, here, scRNA-seq was used to uncover the cell types and reveal their transcriptional signatures, as well as to identify cell-type-specific markers to yield a transcriptional cell atlas of all cell types in the testis.

## 2. Results

### 2.1. Transcriptome Profile of Adult Orange-Spotted Grouper Testis Tissue

**Figure 1:**
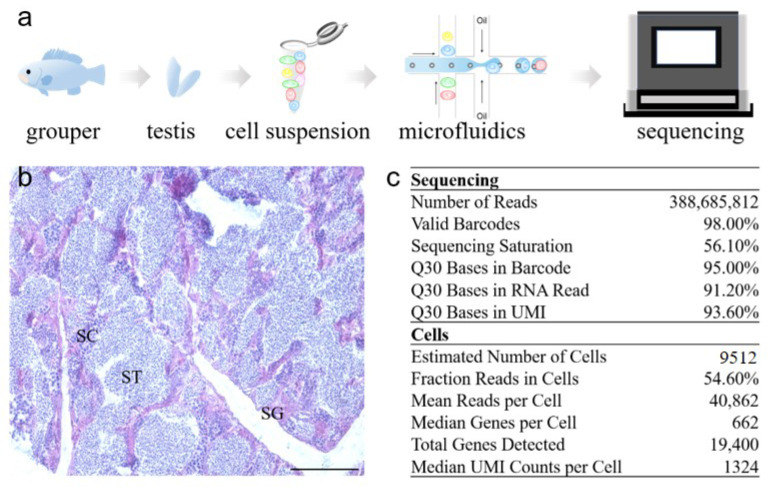
Overview of scRNA-seq experiment design and statistics. (**a**) The workflow of the whole experiment. A male, orange-spotted grouper was obtained, and then the gonad was sampled. Part of the gonad was prepared into single-cell suspension, then every cell was packaged to a single microsphere for subsequent scRNA-seq. (**b**) Gonadal histological morphology of the male grouper by H&E staining. Scale bar = 50 μm. (**c**) The basic information of scRNA-seq data. SG, spermatogonium; SC, spermatocyte; ST, spermatid.

To construct a comprehensive testis map, fresh testis tissue was isolated from the gonad of a male, orange-spotted grouper, and the cell suspension was collected via enzymatic digestion and physical filtering according to our previous experience. After the detection of cell viability and cell number, the suspension was used for scRNA-seq and analysis with the Illumina NextSeq 2500 platform (Figure 1a). The histologic morphology of the mature testis was shown with a majority of STs (Figure 1b). In total, 388,685,812 reads were obtained, and 98.00% of barcodes were valid. In all barcode sequences, the quality score of bases ≥30 accounted for 95.00%. Moreover, Unique Molecular Identifier (UMI) sequences, the quality score of bases ≥30 accounted for 93.60%. Altogether, 9512 cells were isolated and profiled using droplet-based scRNA-seq, 40,862 reads per cell were detected and the median number of genes detected per cell was 622 (Figure 1c, Table S1).

### 2.2. Validation of the Cell Types in scRNA-seq Data

**Figure 2:**
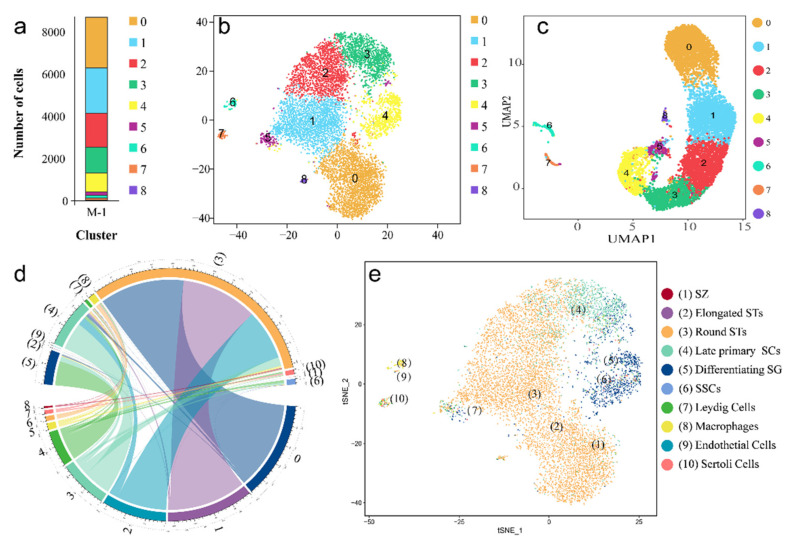
Cell clustering and annotation. (**a**) Distribution of cell number in each cluster. (**b**) Sequenced single cell clusters identified by t-SNE. Each dot represents a cell. (**c**) Sequenced single cell clusters identified by UMAP. (**d**) Distribution of each cluster compared with the ten cell types of human adult testis. The data was visualized via Circos software. 0–8, cluster 0–8 in orange-spotted grouper; (1)–(10), ten cell types in human testis. (**e**) The distribution of each cell types after comparing with the human testis visualized by t-SNE. SSC, spermatogonia stem cell; SG, spermatogonium; SC, spermatocyte; ST, spermatid; SZ, spermatozoon.

Two dimensionality reduction techniques, t-SNE and UMAP, are powerful tools to present the datasets in a more visualized way [16,17]. As shown in Figure 2a, nine clusters of cells were obtained with different numbers. Among all clusters, the summation of clusters 0, 1, 2, and 3 accounted for 84.87% in all cells (Table S2). The t-SNE and UMAP plots showed the same cell numbers and a similar developmental trajectory (Figure 2b,c).

To identify the cell types quickly and accurately, *singleR* was used to annotate the cell types based on human testis scRNA-seq data (GEO: GSE120508) [9,18]. The annotation result showed that all cells in the grouper testis completely matched human testis cells. However, more than one cluster mapped to single cell types in the human data (Figure 2d, Table S3). For example, the cells in cluster 2 aligned with (3) round STs and (4) late primary SCs, and the cells in cluster 7 aligned with (7) Leydig cells, (8) macrophages, (9) endothelial cells, and (10) Sertoli cells. Meanwhile, the t-SNE plot exhibited the detailed distribution of different cell types in grouper testis cells (Figure 2e). Therefore, depending on the annotation result, we re-clustered the cells to 10 types including (1) SZ, (2) elongated STs, (3) round STs, (4) late primary SCs, (5) differentiating SCs, (6) SSCs, (7) Leydig cells, (8) macrophages, (9) endothelial cells, and (10) Sertoli cells.

### 2.3. Validation of the Known Marker Genes in Re-Clustering Clusters

**Figure 3:**
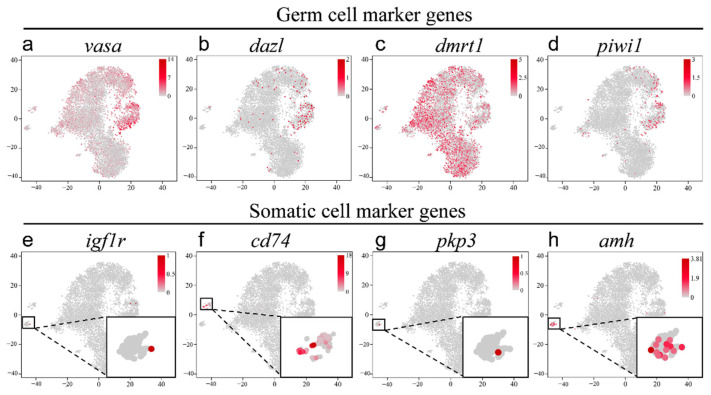
The known markers located in all clusters visualized by t-SNE. (**a**–**d**) The germ cell marker genes. (**a**) *vasa*, (**b**) *dazl*, (**c**) *dmrt1*, (**d**) *piwi1*. (**e**–**h**) The somatic cell marker genes. (**e**) *igf1r*, (**f**) *cd74*, (**g**) *pkp3*, (**h**) *amh*.

Furthermore, based on the expression of known cell-type markers, all cells were divided into two groups, germ cells and somatic cells (Figure 3). Germline-specific markers were expressed solely in clusters 0–5 (e.g., *vasa*, *dazl*, and *piwi1*). The expression of *vasa* was detected in clusters 0–5, and especially in clusters 3 and 4, corresponding to late primary SCs and differentiating SG (Figure 3a). Moreover, *dazl* and *piwi1* were highly expressed in clusters 3 and 4 (SG and SCs), which was consistent with our previous results [19] (Figure 3b,d). Moreover, the known male-specific germ cell marker *dmrt1* was highly expressed in almost all germ cells (clusters 0–5, Figure 3c). Next, four genes were used to distinguish the somatic cells. Leydig cells (marked by *igf1r* [20], insulin-like growth factor 1 receptor), a type of mesenchymal cell, are located between SCs with an important role in testicular development, spermatogenesis, and male phenotypes (Figure 3e). Testicular macrophages promote testicular function and spermatogonia maintenance [6,7] and were identified by multiple specific markers (i.e., CD14, CD74, CD163; Figure 3f). Furthermore, *pkp3*, an endothelial cell marker gene, was also expressed in endothelial cells specifically (Figure 3g). Moreover, Sertoli cells support germ cell survival, development, and physiological functioning and are marked by *sox9* (Figure S1) and *amh* (Figure 3h) [2,21]. The expression of four marker genes was consistent with previous annotation results. The specific expression levels of eight known markers in nine clusters are listed in Table S4.

### 2.4. Screening and Identification of New Marker Genes

**Figure 4:**
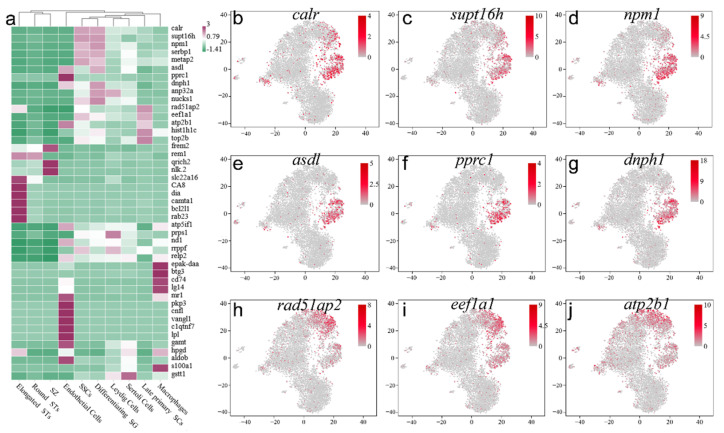
Selection of germ cell marker genes. (**a**) The heatmap of the most DEGs in 10 cell types. (**b**–**j**) The selected genes located in all clusters visualized by t-SNE. (**b**) *calr*, (**c**) *supt16h*, (**d**) *npm1*, (**e**) *asdl*, (**f**) *pprc1*, (**g**) *dnph1*, (**h**) *rad51ap2*, (**i**) *eef1a1*, (**j**) *atp2b1*. DEGs, differential expressed genes. SSC, spermatogonia stem cell; SG, spermatogonium; SC, spermatocyte; ST, spermatid; SZ, spermatozoon.

**Figure 5:**
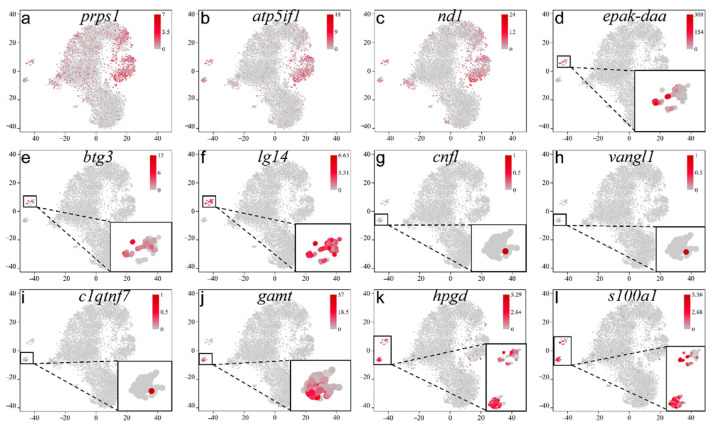
The selected marker genes located in all clusters visualized by t-SNE. (**a**) *prps1*, (**b**) *atp5if1*, (**c**) *nd1*, (**d**) *epak-daa*, (**e**) *btg3*, (**f**) *lg74*, (**g**) *cnfl*, (**h**) *vangl1*, (**i**) *c1qtnf7*, (**j**) *gamt*, (**k**) *hpgd*, (**l**) *s100a1*.

To find more markers suitable for orange-spotted grouper, the top five DEGs in ten cell types were selected (Figure 4a) and their corresponding t-SNE plots are shown according to the re-clustering result (Figure 4b–j and Figure 5). The specific expression levels of top five DEGs in nine clusters were listed in Table S5. Depending on the result, *calr*, *supt16h*, and *npm1* were the top three DEGs in SSCs with relatively high expression in SG and a small number of SCs (Figure 4b–d). *Pprc1*, *asdl*, and *dnph1* were expressed in SG specifically (Figure 4e–g), and *rad51ap2*, *eef1a1*, and *atp2b1* were mainly expressed in SCs (Figure 4h–j).

In somatic cells, *atp5if1*, *prps1*, and *nd1* were the top three DEGs in Leydig cells and were also expressed in SG (Figure 5a–c), and *epak-daa*, *btg3*, and *lg14* were expressed in macrophages specifically (Figure 5d–f). *Cnfl*, *vangl1*, and *clqtng7* possessed unique expression in endothelial cells (Figure 5g–i). *Gamt*, *hpgd*, and *s100a1* were expressed in Sertoli cells specifically (Figure 5j–l).

**Figure 6:**
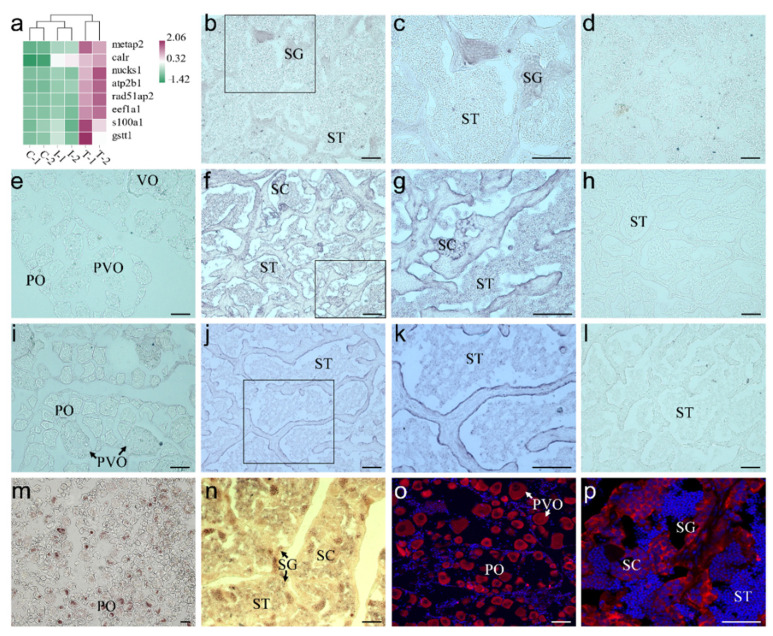
Validation of the selected marker genes. (**a**) Heatmap of eight selected marker genes in previous RNA-seq data. (**b**) *Calr* mRNA expression in testis, (**c**) a higher magnification view of the box area in (**b**,**d**) negative contrast hybridized by *calr* sense probes. (**e**) *Eef1a* mRNA expression in ovary, (**f**) *eef1a* mRNA expression in testis, (**g**) a higher magnification view of the box area in (**f**,**h**) negative contrast hybridized by eef1a sense probes. (**i**) *S100a1* mRNA expression in ovary, (**j**) *s100a1* mRNA expression in testis, (**k**) a higher magnification view of the box area in (**j**,**l**) negative contrast hybridized by *s100a1* sense probes. (**m**) *Vasa* mRNA expression in ovary, (**n**) *vasa* mRNA expression in testis, (**o**) Vasa antibody expression in ovary, (**p**) Vasa antibody expression in testis. PO, primary-growth stage oocyte; PVO, cortical-alveolus stage oocytes; VO, vitellogenic stage oocytes; SG, spermatogonium; SC, spermatocyte; ST, spermatid; SZ, spermatozoon. Scale bar = 50 μm.

For the 50 candidate markers, we attempted to analyze their expression in our previous RNA-seq data (DDBJ: PRJDB9134). The data showed that eight genes were expressed at higher levels in the testis more than in the natural ovary and the gonad in the early stage of sex reversal (Figure 6a, Table S6). Differently expressed in the ovary and testis, three of eight genes were verified based on their expression profiles in different gonads. Therefore, ISH was used to validate the expression profiles of the candidate marker genes in the gonad. The primers for ISH were listed in Table 1. In the natural male section of orange-spotted grouper, ISH results indicated that *calr* was only detected in SG without any signals in other cells (Figure 6b,c). Moreover, a sense probe of *calr* did not provide signal in the natural testis section (Figure 6d). Meanwhile, *eef1a* mRNA signals were observed in SG, SCs, and SZ with the highest expression in SCs (Figure 6f,g), which was consistent with our scRNA-seq data. Furthermore, eef1a was not detected in the ovary (Figure 6e), and the sense probe also resulted in no signals (Figure 6h). Additionally, *s100a1* was detected in the natural ovary and testis, but there were no signals in the ovary, SG, SCs, and STs, except in Sertoli cells (Figure 6i–k). The sense probe of *s100a1* had no signals, too (Figure 6l). In addition, the location of its RNA and protein of *vasa*, as a conservative germ cell marker, was validated in the ovary and testis. *Vasa* RNA was highly expressed in the primary-growth stage oocyte, and the signals were condensed in the Balbiani body (Figure 6m). In the primary-growth stage oocyte, vasa RNA signals were uniformly dispersed in the cytoplasm. In the testis, vasa RNA signals are mainly distributed in all germ cells with higher expression in SG (Figure 6n). However, the location of Vasa protein exhibited some differences from the ISH result. Vasa was expressed in all oocytes evenly (Figure 6o), SG and SCs, but not STs (Figure 6p).

### 2.5. Pseudotime Trajectory of Spermatogenesis in Adult Orange-Spotted Grouper

**Figure 7:**
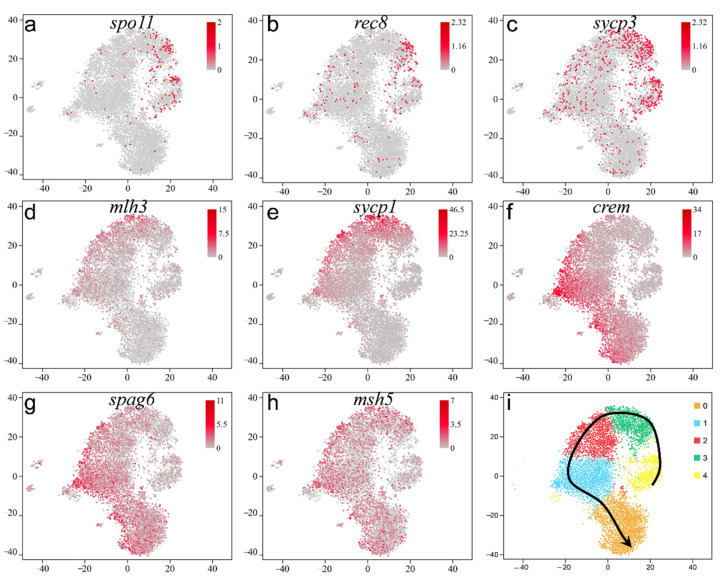
Developmental trajectory of male germ cells. (**a**) *spo11*, (**b**) *rec8*, (**c**) *sycp3*, (**d**) *mlh3*, (**e**) *sycp1*, (**f**) *crem*, (**g**) *spag6*, (**h**) *msh5*, (**i**) the developmental trajectory of male germ cells (cluster 0–4). From cluster 0 to cluster 4, spermatogonia differentiates into sperm gradually. (**b**−**i**), meiosis-related genes located in all clusters visualized by t-SNE.

Many specific expressed genes were selected to profile the process of spermatogenesis. In our data, *spo11*, *rec8*, and *sycp3* were highly expressed in primary SCs (Figure 7a–c), and *mlh3* and *sycp1* were also highly expressed late primary SCs (Figure 7d,e), which was distinctly different from the location of *spo11*, *rec8*, and *sycp3* in the t-SNE plot. However, *msh5* was localized to in almost all germ cell and not limited to SCs (Figure 7h), which is different from the function in mammals [22]. Moreover, *crem* and *spag6*, encoding ST structural proteins, were confined to STs (round STs and elongated STs; Figure 7f,g).

Depending on the UMAP plot and the expression of meiosis-related genes and ST structure-related genes, we plotted the pseudotime trajectory of male germ cells in orange-spotted grouper (Figure 7i). SSCs (cluster 4) differentiate into SG (cluster 4), and then, SG begin to differentiate into early primary SCs. Primary SCs continue to differentiate in to late primary SCs (cluster 3). Late primary SCs develop into round STs (clusters 0–2) and elongated STs (cluster 0). Finally, STs transform into sperm (cluster 0).

### 2.6. Adult Male Germ Cell Development in Orange-Spotted Grouper

**Figure 8:**
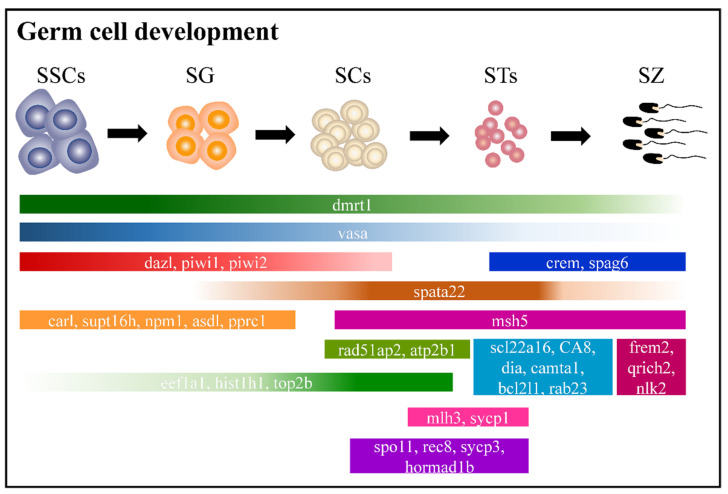
Male, orange-spotted grouper spermatogenesis model. Black arrows indicate the direction of differentiation. The expression pattern of key marker genes (both previously known and discovered in this study) are indicated by boxes. SSCs, spermatogonia stem cells; SG, spermatogonium; SC, spermatocyte; ST, spermatid; SZ, spermatozoon.

Spermatogenesis includes three stages: (1) spermatogonia proliferate and differentiate to produce primary spermatocytes; (2) primary SCs produce STs via two meiosis events; (3) STs form mature sperm after metamorphosis [23]. Combined with the known and new-found markers, the blueprint of adult male germ cell development was plotted with many cell-specific and stage-specific markers in orange-spotted grouper (Figure 8).

## 3. Discussion

Adult spermatogenesis is a complex process in grouper, and full data of spermatogenesis is limited. Here, we aimed to provide foundational scRNA-seq data of all cells contained within the natural male adult testis of orange-spotted grouper, complemented by computational analysis and validation studies—to offer a detailed map of male gametogenesis in groupers. Previously, LCM had been used to obtain male germ cells of spermatogenesis in orange-spotted grouper, which resulted in a profile of changes in male germ cells and the characterization of cell markers [15]. However, the whole process of LCM must produce a sufficient amount of RNA with high quality to ensure the reliability of transcriptome results. We had to consider that single-cell LCM requires a long microdissection period, and the yield of RNA is limited. Consequently, we moved our focus to scRNA-seq, which has recently been applied to study spermatogenesis, because of its high throughput and accuracy for experiments on many mammals [9,10,11,12]. In contrast to the traditional transcriptome analysis of tissue, a single-cell transcriptome overcomes the homogenization of various cell types in the same tissue and exposes the molecular heterogeneity of every single cell, which provides a comprehensive profile and significant novel biological insights [24]. Therefore, scRNA-seq was used to profile the spermatogenesis of orange-spotted grouper.

Grouper, widely distributed throughout the tropical and subtropical waters of the world, is regarded as a favorite marine food fish. With a lack of natural males, large-scale aquaculture of grouper is limited. The gonadal development of the grouper will undergo a transition from ovary to intersexual gonad and then to testis [25]. Due to this reproductive characteristic, we cannot directly distinguish the sexuality of orange-spotted grouper by morphology. Therefore, we selected the male grouper by squeezing the belly softly. If white liquid (semen) flowed from the cloacal aperture, the adult fish was considered a mature male. That is why the number of spermatids was 90% of all cells in our scRNA-seq data; simultaneously, SG and SCs comprised a small proportion of male germ cells. Even so, we identified six kinds of germ cells and four kinds of somatic cells, which included enough information of the testis without other influences for further analysis.

Moreover, many marker genes were found in germ cells and somatic cells. Two well-known germ cell markers, *vasa* and *dazl*, had no identical expression in testis (Figure 3a,b). Due to its similar structure and function to the prototypical DEAD-box helicase eukaryotic translation initiation factor 4A (eIF4A), Vasa is a member of the DEAD-box helicase family, also known as DDX4, which are responsible for RNA binding, ATP binding, and ATP hydrolysis [26,27]. However, in the bipotential gonad, Vasa mRNA and its protein, appear to be continuously present in the germline from the beginning of germ-cell specification throughout germ-cell maturation, persisting to the mature ovary and testis [28,29,30]. Moreover, with our present result, *vasa* mRNA and its protein persisted in PGCs (unpublished data), oocytes, male germ cells, and germ cells in the intersexual gonad (Figure 6m–p). Therefore, vasa, a germ cell marker, is the most conservative and stable gene in orange-spotted grouper so far. The Deleted in Azoospermia (DAZ) gene family, which includes *daz*, *dazl*, and *boule*, encodes germ cell-specific RNA-binding proteins that are implicated in the translational regulation of several transcripts [31,32,33]. However, *dazl* was not expressed in all male germ cells, especially in STs and sperms, which is consistent with the location of *dazl* in Asian seabass [34]. Moreover, a specific class of Argonaute proteins, Piwi proteins, is also important in germ cell development [35]. *Piwi1* possessed similar expression pattern with those of *dazl*.

Combined with our previous RNA-seq [36] and scRNA-seq data, several marker candidates (*calr*, *eef1a*, *s100a1*) were selected and identified. Calreticulin (Calr), a highly conserved endoplasmic reticulum chaperone protein, ensures proper protein conformation and prevents protein aggregation [37,38,39]. It was reported that Calr may take part in the process of wound healing via TGF-β [40]. However, the relationship between Calr and reproduction is unclear, especially in teleost. The unique location indicated that *calr* may participate in the proliferation of SG, which provides new insight into the spermatogenesis of the orange-spotted grouper. Eukaryotic translation elongation factor 1A, eEF1A, is one of the most abundant protein synthesis factors. EEF1A plays important roles in various biological processes, including reconstruction of the cellular skeleton, folding and degradation of proteins, regulation of cell signals, and cell growth/apoptosis, among others [41,42,43,44]. Therefore, the expression of eEF1A in the testis might be related to spermatogenesis, especially in SCs. Binding with Ca^2+^ and other target proteins, S100a1 regulates a wide variety of physiological processes, including cell apoptosis, cell differentiation, and gene transcription [45,46,47], etc. Located in Sertoli cells, S100a1 attracted our interest for further investigation of its role in spermatogenesis in the orange-spotted grouper.

In the entire process of spermatogenesis, there are two successive meiosis events—the first meiosis is of the primary SCs and the second meiosis is of the secondary SCs. Meiosis is an important process during sexual reproduction, and the exchange of chromatids during meiosis not only enhances the genetic diversity of the species but also provides more resources for creating a new germplasm [48]. In meiosis, DNA replicates once and SCs divide twice. The correct completion of meiosis ensures the stable transmission of genetic information and diversity of the species. In the first meiotic division, homologous chromosomes undergo a complex sequence of events, including pairing, association, recombination, and separation [49]. In the second meiotic division, the sister chromatids separate, eventually forming haploid cells (STs or oocytes). In mammals, Spo11, a topoisomerase, initiates meiotic recombination after DNA replication at the early stage of first meiosis. The meiotic cohesion factor, Rec8 can dismantle centromeres [50]. The topoisomerase complex and other factors catalyze the breakage of double-stranded DNA, following which the 3′ terminal of single stranded DNA (ssDNA) is exposed. Mlh3, mismatch-repair protein, participates in the recombination of ssDNA [51]. The naked ssDNA is integrated with nucleoprotein filaments by Rad51 and Dmc1 [52]. After the repair of DNA double-strand breaks, Zip1– 3 proteins, Msh4/Msh5 [8], Sycp3, and paired homologous chromosomes form the synaptonemal complex [53]. In the pachytene stage, Sycp1 participates in the recombination of homologous chromosomes [54].

Furthermore, we selected many marker candidates of other germ cells and somatic cells. Depending on the location of these genes, we plotted a map of adult male germ cell development in orange-spotted grouper, which is beneficial for cell identification. In the future, we aim to focus more effort on these genes to reveal their detailed roles in spermatogenesis.

## 4. Materials and Methods

### 4.1. Animal

Adult male, orange-spotted grouper was raised in the Guangdong Daya Bay Fishery Development Center (Huizhou 516081, Guangdong, China). The fish were kept in indoor pools, and the water temperatures were controlled at 22.7~27.8 °C. From our previous experience, we expected the cloacal aperture of the adult male to outflow white seminal fluid when we pushed the abdomen of the fish gently. Thus, we selected an adult male (length = 63 cm, weight = 5.62 kg), and the testis was sampled to confirm the developmental stages and prepare single-cell suspensions after anesthetized with MS222. All animal experiments were conducted following the guidelines and approval of the respective Animal Research and Ethics Committees of Sun Yat-Sen University.

### 4.2. Hematoxylin-Eosin (H&E) Staining

After fixed in Bouin’s solution for 24 h, the testis was orderly dehydrated using gradient alcohol. Then, the testis was embedded by paraffin until transparent, then dealt with xylene. The paraffin blocks were cut into slices with 5 μm using a paraffin slicing machine (Lecia, Wetzlar, Germany). Finally, the nucleus and cytoplasm were stained by hematoxylin and eosin, respectively, and the sections were observed by light microscopy (Nikon IQ50, Tokyo, Japan) to classify the gonadal stages.

### 4.3. The Preparation of Gonadal Single-Cell Suspension

The gonad of male, orange-spotted grouper was dissociated for single-cell transcriptomics. Gonad was cleaned by 1× Hank’s balanced salt solution twice to remove the erythrocyte and other impurities associated with gonad. Next, the sample was cut into pieces, then centrifuged at 100× *g* for 3 min and incubated with 500 U/mL pancreatin (without calcium and magnesium ion) at 37 °C for 40 min. Then, the cell suspension was centrifuged at 100× *g* for 3 min and filtrated by a 40 µm strainer (Corning Inc., Corning, NY, USA). The cell number and motility rate of the suspension were calculated by the blood counting chamber.

### 4.4. Single Cell RNA-seq Library Construction and Sequencing

ScRNA-Seq was performed using the 10× Genomics system. According to manufacturer recommendations, loaded into 10× Chromium Controller using Chromium Single Cell 3′ v2 reagents, cells were diluted and mixed with 33.8 µL of the total mixed buffer. After 13 cycles for cDNA amplification, ~100 ng of cDNA was used for library amplification by 12 cycles. The resulting libraries were then sequenced on a 26 × 100 cycle paired end run on an Illumina HiSeq 2500 instrument.

### 4.5. Process of Single Cell RNA-seq Data

A Cell Ranger v1.2.1 was used to process raw sequencing data, then generate three types of fastq files running with Cell Ranger v1.2.1, including I1 containing the 8 bp sample index; R1 contains 26 bp (10 bp cell-BC + 16 bp UMI) index and R2 contains 100 bp cDNA sequence. Then, the data were extracted using alignment filtering and cellular barcode and UMI counting.

### 4.6. Cell Clustering and Trajectory Analysis

To cluster the cells, the modularity optimization technique–SLM [55] was applied to iteratively group cells together and optimize the standard modularity function. Seurat continues to use t-distributed Stochastic Neighbor Embedding (t-SNE) [16] as a powerful tool to visualize and explore these datasets. The t-SNE aims to place cells with similar local neighborhoods in high-dimensional space together in low-dimensional space. Besides, another dimensionality reduction technique, Uniform Manifold Approximation and Projection (UMAP), was used to plot clusters in a developmental time-course in a meaningful continuum of clusters along a trajectory [56]. The final number of clusters was decided by the specific gene markers and the relative relationships with other clusters.

Single-cell trajectory was analyzed using a matrix of cells and gene expressions by Monocle (Version 2.6.4). Monocle reduced the space down to one with two dimensions and ordered the cells (sigma = 0.001, lambda = NULL, param.gamma = 10, tol = 0.001). Once the cells were ordered, we could visualize the trajectory in the reduced dimensional space with a tree-like structure, including tips and branches.

### 4.7. Differential Expression Analysis

Genes usually interact with each other to play roles in certain biological functions. Significantly enriched Gene Ontology (GO) terms were selected by Q value (Q < 0.05). KEGG (Kyoto Encyclopedia of Genes and Genomes) is the major public pathway-related database helping to further understand the gene’s biological functions. The *p*-value was gone through FDR Correction, taking FDR ≤ 0.05 as a threshold. Pathways meeting this condition were defined as significantly enriched pathways in differentially expressed genes (DEGs).

### 4.8. In Situ Hybridization (ISH)

The protocol of ISH was referred to a previous study, but with minor modifications [57]. Briefly, fresh gonad samples were fixed with 4% paraformaldehyde in PBS (0.1% DEPC water dilute) at 4 °C overnight. The samples were dehydrated in 30% sucrose at 4 °C overnight and then embedded with OCT (SAKURA Tissue-Tek^®^, Torrance, CA, USA). Testis and ovary blocks were cryosectioned at 5 μm and 6 μm, respectively. The sections were mounted on superfrost plus microscope slides (Thermo, Waltham, MA, USA) for ISH. cDNA fragments of genes were inserted into pGEM-EASY vector for synthesizing the sense and anti-sense digoxigenin (DIG) labeled riboprobes using the RNA DIG Labeling Kit (Roche, Mannheim, Germany). The samples were hybridized by DIG-labeled RNA probes in 58 °C oven. After hybridization, the sections were washed by gradient SSC buffers and PBS buffer. The DIG label was tested with an alkaline phosphatase-conjugated anti-DIG antibody (Roche Diagnostics; diluted 1:1000) and colored the signal with NBT/BCIP Stock Solution (Roche Diagnostics). Finally, the sections were mounted with the water-soluble reagent (Boster, Wuhan, China) and imaged with a microscope (Nikon IQ50, Tokyo, Japan).

### 4.9. Immunofluorescence (IF)

The protocol of IF was referred to our previous experience, but with minor modifications [36]. Briefly, the gonad from a male, orange-spotted grouper was sampled and then embedded for the frozen section. The frozen gonadal sections were incubated with the first antibody of Vasa (Rabbit anti-Vasa [58], 1:100 diluted with 2% BSA in PBS) and the second antibody HRP-conjugated anti-mouse IgG (Boster, Wuhan, China) at a dilution of 1: 2000 with 2% goat serum in PBS. Fluorescence signals were detected by the TSATM Plus Fluorescence System (Roche, Basel, Switzerland) and the cell nuclei were stained by 4′,6-diamidino-2-phenylindole (DAPI). Finally, the sections were observed under a confocal fluorescence microscope (Leica, TCS-SP5, Wetzlar, Germany).

## 5. Conclusions

Our present study was the first comprehensive transcriptional atlas of the male testis at a single-cell level in teleosts, and especially hermaphrodite fish. We identified every cell type in the testis and defined comprehensive cell-specific gene signatures, which also provides a foundation for the identification of male germ cells, and which will help us to explore the origin of male germ cells during sex reversal. Our findings offer new insight into understanding the delicate process of spermatogenesis in the hermaphroditic, orange-spotted grouper.

## Figures and Tables

**Table 1:** The primers used in the present study.

Primers	Purpose	Sequence (from 5′ to 3′)
*calr*-F	ISH	GACGCCACCGTCTACTTCAA
*calr*-R	ISH	GTCGTCCCAGTCACTTGGTT
*eef1a*-F	ISH	AAGGGCTGGAAGATCAACCG
*eef1a*-R	ISH	TTAATCACTCCCACGGCCAC
*s100a1*-F	ISH	AAAGCCCAGAAGAACCCCAA
*s100a1*-R	ISH	CGACGAGGGGAAGAAACTCT
*vasa*-F	ISH	GAGCCTGAGACCATCATC
*vasa*-R	ISH	AGGACTCTTCACACTGTTG

## Data Availability

In present study, scRNA-seq data can be obtained from the Genome Sequence Archive under accession number CRA004714. RNA-seq data can be obtained from the Transcriptome Shotgun Assembly project DDBJ under accession number PRJDB9134.

## Supplementary Materials

The following are available online at https://www.mdpi.com/article/10.3390/ijms222212607/s1.
