# Supplementary figures and images for: Single-Cell Atlas of Adult Testis in Protogynous Hermaphroditic Orange-Spotted Grouper, Epinephelus coioides
Source: Int J Mol Sci. 2021 Nov 22;22(22):12607. doi: 10.3390/ijms222212607 (PMC8618070; doi:10.3390/ijms222212607)

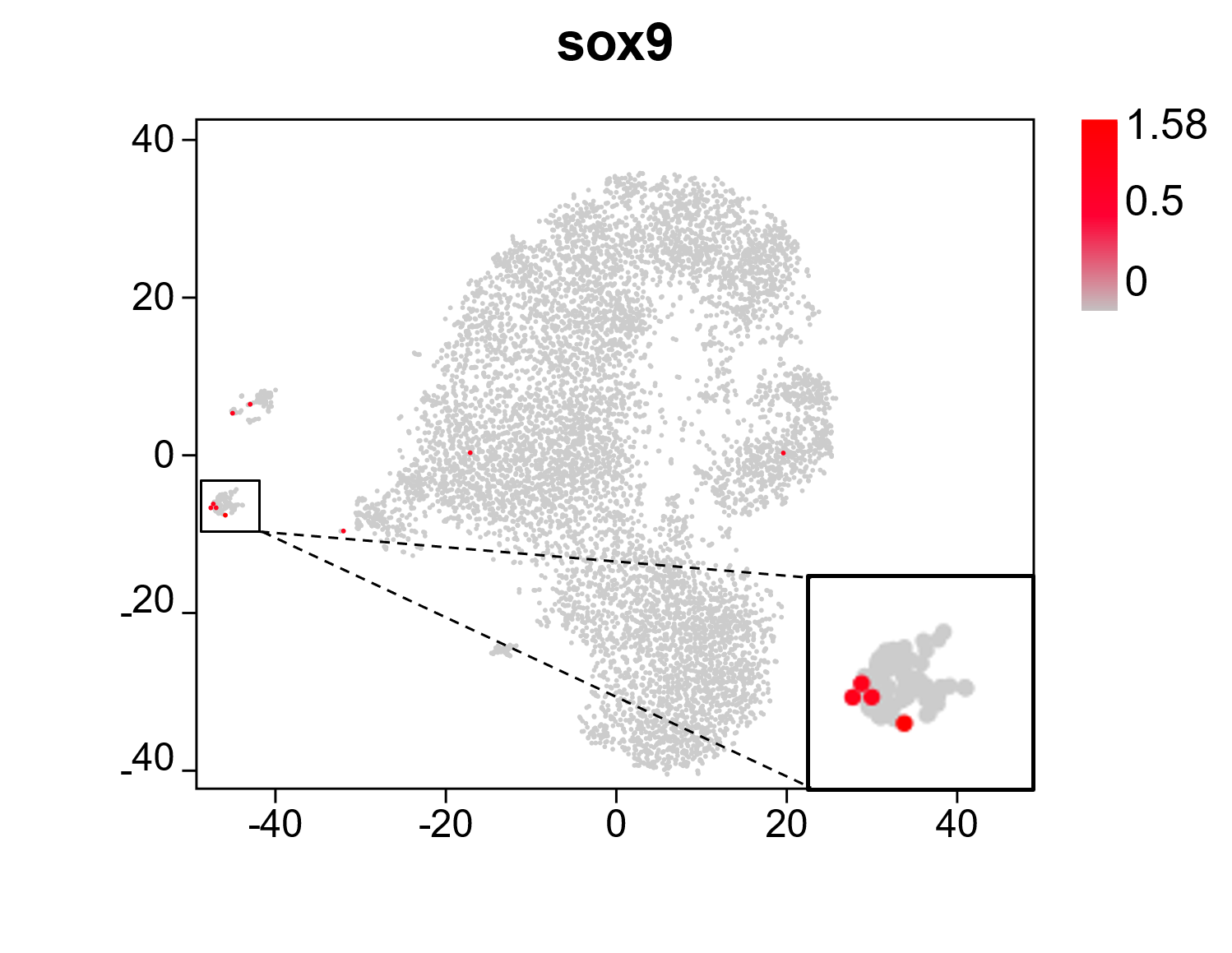

Supplement: Supplementary file 1 [file ijms-22-12607-s001.zip › supplemental files/2 Figure S1.tif]
